# Single versus two-stage management of long-bone chronic osteomyelitis in adults: a systematic review and meta-analysis

**DOI:** 10.1186/s13018-024-04832-7

**Published:** 2024-06-14

**Authors:** Ali Lari, Ali Esmaeil, Matthew Marples, Arun Watts, Bethan Pincher, Hemant Sharma

**Affiliations:** 1 Al-Razi Orthopedic Hospital, Kuwait, Kuwait; 2 Hull University Teaching Hospitals, Hull, UK; 3 Hull York Medical School, University of Hull, Hull Limb Reconstruction & Bone Infection Unit, Hull University Teaching Hospitals, Hull, UK

**Keywords:** Osteomyelitis, Single stage, Two-stage, Bone infection, Debridement, Reconstruction

## Abstract

**Background:**

Chronic osteomyelitis is a debilitating bone infection, characterized by a persistent infection over months to years, poses diagnostic and therapeutic challenges due to its insidious nature and potential for severe bone and soft tissue destruction. This systematic review and meta-analysis aims to review the literature on the treatment of chronic osteomyelitis in long bones and assess cure rates in single versus two-stage surgery.

**Methods:**

Following the PRISMA guidelines and registered with PROSPERO (ID: CRD42021231237), this review included studies that reported on the management of chronic osteomyelitis in long bones using either a planned one-stage or two-stage surgical approach in adult patients. Databases searched included Medline, Embase, Web of Science, CINAHL, HMIC, and AMED, using keywords related to osteomyelitis, long bones, and surgical management. Eligibility criteria focused on adults with chronic osteomyelitis in long bones, with outcomes reported after a minimum follow-up of 12 months. The meta-analysis utilized the random-effects model to pool cure rates.

**Results:**

The analysis included 42 studies with a total of 1605 patients. The overall pooled cure rate was 91% (CI 95%) with no significant difference observed between single-stage and two-stage surgeries (X2 = 0.76, P > 0.05). Complications were reported in 26.6% of cases in single-stage procedures and 27.6% in two-stage procedures, with prolonged wound drainage noted as a common issue. Dead space management techniques varied across studies, with antibiotic-loaded calcium sulphate beads used in 30.4% of cases.

**Conclusion:**

This meta-analysis reveals no significant difference in cure rates between single and two-stage surgical treatments for chronic osteomyelitis in long bones, supporting the efficacy of both approaches. The current treatment strategy should include a combination of debridement, dead space management using local and systematic antibiotics and soft tissue reconstruction if necessary.

## Introduction

Osteomyelitis is characterized by an infectious and destructive inflammatory process affecting the bone that stems from microorganisms' invasion. The infection's etiology varies, originating either from local spread linked to trauma and surgery or from hematogenous dissemination, particularly in the elderly and children [1, 2]. The disease is often compounded by immune, vascular, and soft tissue problems [2]. The manifestations of chronic osteomyelitis are diverse, often remaining indolent for months before symptoms become apparent. The distinction between acute and chronic osteomyelitis, however, is contentious [3]. While some define chronicity based on histopathological examination and sequestrum formation, others consider it chronic when the infection persists for months to years, an arbitrary but commonly used timeframe [1–4]. Nevertheless, chronic osteomyelitis evolves over an extended period, potentially leading to sequestrum, bone destruction, marrow infection, soft tissue involvement, and fistulous tracts [4]. The severity can vary widely, from simple, manageable infections to severe cases with extensive bone destruction, significant functional deficit and even limb loss.

The management of osteomyelitis requires a multifaceted and aggressive approach to eradicate the infection and optimize outcomes [5–7]. Treatment modalities vary, and decision-making remains challenging as it encompasses various surgical techniques, antibiotic delivery methods, duration of antibiotic treatment, and surgical staging [7]. The problems the patient may encounter are multifaceted, and has been highlighted in the classification by Cierny and Mader et al., which categorizes osteomyelitis based on anatomical location, physiological status, and high-risk factors [8]. The complexity of treating osteomyelitis depends on its location, often involving long-term and debilitating treatment regimens. Success is typically indicated by a prolonged remission period, but conclusively declaring the disease cured is often problematic due to late recurrence.

Traditionally, treatment has relied on prolonged antibiotic use and multiple surgical debridements. In two-stage procedures, the primary focus is on eliminating the infection through bone and soft tissue resection, followed by stabilization of the bone, often externally, using fixators or frames. A second stage is planned approximately 4–8 weeks later, though this period can vary. This stage occurs after a course of antibiotics and once the infection has resolved both clinically and biochemically. The second stage concentrates on restoring function, utilizing techniques like fibular grafts, the Masquelet technique, autologous cancellous bone grafts, or bone transport [9–11]. Conversely, single-stage techniques aim to eradicate the infection with appropriate debridement and both local and systemic antibiotics, managing the bone defect in the same stage using techniques similar to those used in the second stage of two-stage management [12–14].

Current approaches emphasize a single thorough debridement, effective management of dead space, both local and systemic antibiotic administration, and a multidisciplinary strategy [15–17].

Comparisons between these techniques in the literature are scarce. This systematic review and meta-analysis aim to thoroughly review the literature on the treatment of chronic osteomyelitis in long bones and assess cure rates in single versus two-stage surgery for the condition.

## Methods

The search and selection process followed the Preferred Reporting Items for Systematic Reviews and Meta-Analyses (PRISMA) guidelines and was prospectively registered with PROSPERO (International Prospective Register of Systematic Reviews) (ID: CRD42021231237).

### Search strategy

A systematic search of Medline, Embase, Web of Science, CINAHL (Cumulative Index to Nursing and Allied Health Literature), Healthcare Management Information Consortium (HMIC) and the Allied and Complementary Medicine (AMED) databases was performed using the following search strategy: (("osteomyelitis"[Title/Abstract] OR "bone infection"[Title/Abstract]) AND ("humerus"[Title/Abstract] OR "ulna"[Title/Abstract] OR "radius"[Title/Abstract] OR "fibula"[Title/Abstract] OR "single stage"[Title/Abstract] OR "one stage"[Title/Abstract] OR "two stage"[Title/Abstract] OR "two-stage"[Title/Abstract] OR "long bone"[Title/Abstract] OR "femur"[Title/Abstract] OR "tibia"[Title/Abstract])). Finally, reference lists of relevant articles were reviewed to identify additional articles that were potentially missed during the initial search.

### Eligibility criteria

Studies that met the following criteria were included: (1) Management of chronic osteomyelitis in long bones using a planned one-stage or planned two-stage, 2) Patients aged 18 or older, 3) Follow up of at least 12 months, and 4) Clinical outcomes were reported.

Studies were excluded if they met any of the following criteria: (1) Review articles; (2) full text not available; (3) cadaveric studies; (4) Patients less than 18 years; (5) treatment of septic or infected non-unions, (6), non-bacterial osteomyelitis, (7) case series with fewer than 10 patients, (8) joint infections, (9) articles including non-long-bone osteomyelitis were excluded if they did not report outcome data separately for long bones. In addition, articles reporting outcomes on septic non-unions and osteomyelitis collectively were excluded.

### Study screening

Titles and abstracts were independently screened for relevance by three authors using Covidence (AL, AE and MP) (Covidence systematic review software, Veritas Health Innovation, Melbourne, Australia. www.covidence.org). Potentially relevant articles underwent full-text screening, with any conflicts between the authors being resolved by discussion and consensus with the senior authors (HS).

### Quality assessment and risk of *bias*

Study quality assessment was conducted using the methodological index for non-randomized studies (MINORS) tool. Methodological quality was categorized prior as follows: a score of 0–8 or 0–12 was considered poor quality, 9–12 or 13–18 was considered fair quality, and 13–16 or 19–24 was considered excellent quality, for non-comparative and comparative studies, respectively. For randomized controlled trials, the Cochrane risk of bias-2 (RoB-2) tool was used to assess study quality. Quality assessment measurements are denoted after each study in Table 1.

**Table 1:** Baseline demographics and patient characteristics

Study	Study design	MINORS	Sample size	Meeting criteria	Females	Age (yr)	Follow up (mth)	Stage
Ochsner 1990 [30]	R.cohort	8	25	12	4 (16%)	54 (23–82)	26 (2–60)	One
Philandrianos 1992 [31]	R.cohort	7	11	10	1 (9%)	51.3 (29–82)	40.5 (12–82)	One
Guelinckx 1995 [32]	R.cohort	8	16	10	2 (12.5%)	41 (24–73)	24 (6–120)	One
Pfeiffenberger 1996 [33]	R.cohort	7	28	5	NR	47 (6–83)	66 (24–156)	One
Yamashita 1998 [34]	R.cohort	9	18	13	8 (44.4%)	38.7 (14–77)	55 (24–75)	One
Simpson 2001 [35]	P.comparative	15	50	43	11 (22%)	49 (13–82)	26 (12–48)	One
Kuokkanen 2002 [36]	R.cohort	7	21	16	3 (14.3%)	34 (30–69)	30 (7–78)	One
Hashmi 2004 [37]	P.cohort	10	17	8	0 (0%)	37 (17–53)	75 (56–95)	One
Chang 2007 [38]	R.comparative	13	65	65	29 (44.6%)	39.8 (18–69)	75 (36–334)	One
Rao 2007 [39]	P.cohort	12	51	7	27 (52.9%)	55.4 (17–83)	24.9 (3–53)	One
Khan 2012 [40]	R.cohort	7	20	20	3 (15%)	44.5 (6–73)	22.5 (19–36)	One
Romanò 2014 [41]	R.comparative	14	76	76	27 (25.5%)	45.7 (19–80)	21.8 (12–36)	One
Ferrando 2017 [26]	R.comparative	13	25	20	5 (20%)	49 (16–86)	23 (16–33)	One
Badie 2019 [42]	P.cohort	11	30	30	5 (16.7%)	26.3 (17–53)	min. 12	One
Oosthuysen 2019 [43]	R.cohort	9	24	14	4 (28.6%)	34.8 (16–45)	18.1 (12–29)	One
Zhou 2020 [27]	R.cohort	9	42 (43 limbs)	42	19 (45.2%)	43.7 (23–74)	42.8 (12.8–77.5)	One
Al-Mousawi 2020 [44]	R. Cohort	7	12	12	5 (41.7%)	63 (35–74)	16 (12–24)	One
Hotchen 2020 [45]	P. Cohort	13	71	63	17 (23.9%)	48.8 (19.9–82.9)	Min. 24	One
Lorentzen 2020 [46]	R.cohort	7	11	9	3(27.3%)	62 (39–79)	26.4 (15–42)	One
Bor 2022 [47]	R. cohort	9	16	15	3 (18.6%)	49 (13–71)	72 (18–192)	One
Elhessy 2022 [48]	R. Cohort	9	14	14	4 (28.6%)	43.4 (17–73)	30.1 (20–49)	One
Jagadeesh 2022 [13]	R.Review of P.Data (comparative)	16	100	100	31 (31%)	40.35	32.2 (24–63)	One
Luo 2022 [49]	R.cohort	8	17	16	1 (5.9%)	41.9 (8–70)	> 2 yr	One
McNally 2022 [24]	P.cohort	12	100	70	35 (35%)	51.6 (23–88)	72.6 (50.4–100.8)	One
Jiamton 2023 [50]	P. Cohort	9	62	59	13 (21%)	47.2	12	One
Langit 2023 [51]	R. Cohort	9	53 (54 bones)	53	14 (26%)	45.5	29 (12–59)	One
Sambri 2023 [52]	R. Cohort	10	93	93	25 (26.9%)	40 (4–73)	21 (12–84)	One
Ferguson 2023 [14]	R.Review of P.Data (comparative)	17	359	315	NR	49.6 (16–89)	57 (12–126)	One
Perry 1986 [53]	R.cohort	7	14	8	NR	37.7 (23–59)	14.6 (7–18)	two
McNally 1993 [54]	R.cohort	8	37	37	9	42 (18–75)	49 (12–121)	two
Ueng 1994 [25]	R.cohort	8	13	5	3 (23%)	35 (17–59)	37 (24–54)	two
Emara 2002 [29]	R.cohort	9	20	20	2 (10%)	24 (18–39)	34 (30–48)	two
Alonge 2003 [55]	R.cohort	8	25	20	9 (36%)	22.4 (9–44)	46 (19–80)	Two
Zweifel-Schlatter 2006 [56]	R.cohort	8	14	10	1 (7.7%)	39 (16–69)	31.4 (12–52)	Two
Wu 2007 [57]	R.cohort	8	23	7	7 (30.4%)	48.3 (16–82)	55 (24–156)	Two
Wu 2017 [11]	R.cohort	9	36	36	6 (16.7%)	41 (21–68)	29.5 (21–45)	Two
Yu 2017 [9]	R.cohort	9/	13	13	4 (30.8%)	39 (16–69)	17.8 (12–24)	Two
Qiu 2017 [10]	R. Comparative	16	40	40	7 (17.5%)	37.75 (20–71)	30.6 (18–54)	Two
Buono 2018 [58]	R.comparative	12	24	24	6 (25%)	41 (16–75)	30 (12–144)	Two
Wu 2019 [59]	R. Review of P.data	9	28	28	12 (42.9%)	41 (21–68)	29.5 (24–45)	Two
Finelli 2019 [60]	RCT	**Some Concerns	45	45	7 (15.5%)	34.8 (> 18)	24	Both
Zhou 2021 [12]	R.comparative	15	102	102	7 (6.7%)	38 (17–63)	NR	Both
Aggregates			1861	1605	379 (23%)	Mean: 42.7 ± 8.5	36 ± 18	One stage: 28
Two stages: 12								
Comparing: 2								

*R. (Retrospective), P. (Prospective), ** ROB-2

### Data extraction

Three authors independently extracted relevant data from the included studies to a previously piloted Microsoft Excel spreadsheet (Microsoft, Redmond, Washington, USA). These data included general article information, patient demographic and surgical procedure details, and relevant outcome measures.

### Outcomes

Outcomes included the cure rate (%). A meta-analysis of proportions using the random-effects model was used to pool the cure rate (%) estimates from different studies. Without appropriate data transformation, the accompanying meta-analyses experience threats to statistical conclusion validity [18], such as confidence limits falling outside of the established zero-to-one range and variance instability [19]. While the logit transformation solves the problem of confidence interval estimates falling outside the zero to one range, it does not necessarily resolve the issues regarding variance from extreme proportional datasets. As the double arcsine transformation (Freeman-Tukey transformation) addresses both problems listed above, it is the preferred transformation method and was implemented in the current analysis. Once the meta-analysis had been performed on the transformed proportions, a back-transformation was performed. There is still no consensus about the back-transformation method that should be used with the Freeman-Tukey double arcsine method, although the harmonic mean was suggested for back-transformation [20]. Secondary outcomes included types of treatments used, complications, dead space management techniques, length of hospital stay, data on cost and the need for secondary interventions.

### Meta-analysis

Statistical analysis was performed using R v 3.6.3 (R Core Team, Vienna, Austria). The random-effects model (using the maximum likelihood estimator for tau) was used to pool the effect sizes from the included studies. The underlying hypothesis for adopting the random-effects model is that heterogeneity or observed variance of effect is a sum of sampling error and variation in true-effect sizes stemming from inter-population variability. The generic inverse variance method was used to weigh each trial’s per-protocol population. Subgroup analysis was performed based on the stage. The overall proportion was calculated as well as the proportion within each subgroup. Forest plots were used to visualize the results. P values < 0.05 were considered statistically significant.

### Prediction interval

The prediction interval was used to assess the treatment effect that may be predicted in future analyses, considering the different settings across different studies. It captures the variability in the true treatment effect across different settings. With substantial heterogeneity, prediction intervals will be broader than confidence intervals and might be considered a more conservative technique to integrate uncertainty in the analysis [21].

### Sensitivity analysis

Sensitivity analysis was performed using the leave-one-out method to assess the effect of the different studies on the estimate and heterogeneity. Sensitivity analysis was performed to assess whether the pooled estimate and between-study heterogeneity were significantly affected by the exclusion of certain studies.

### Publication bias and heterogeneity between studies

Funnel plots were used to assess publication bias. Egger’s test was used to test the asymmetry of funnel plots [22]. The trim-and-fill method was also used to detect and adjust for publication bias [23]. The I^2^ statistic was used to explore the percentage of heterogeneity attributed to variation in true-effect sizes secondary to inter-population variation. Estimates from subgroups within the same study were pooled using a fixed-effects model and used in the meta-analysis. The 95% confidence interval (CI) and Z-statistic were calculated and used for hypothesis testing. Heterogeneity between studies was quantified using the I^2^ statistic. In the case of high heterogeneity, the cause was investigated, the outlier was removed, and a new result was presented.

## Results

**Fig. 1:**
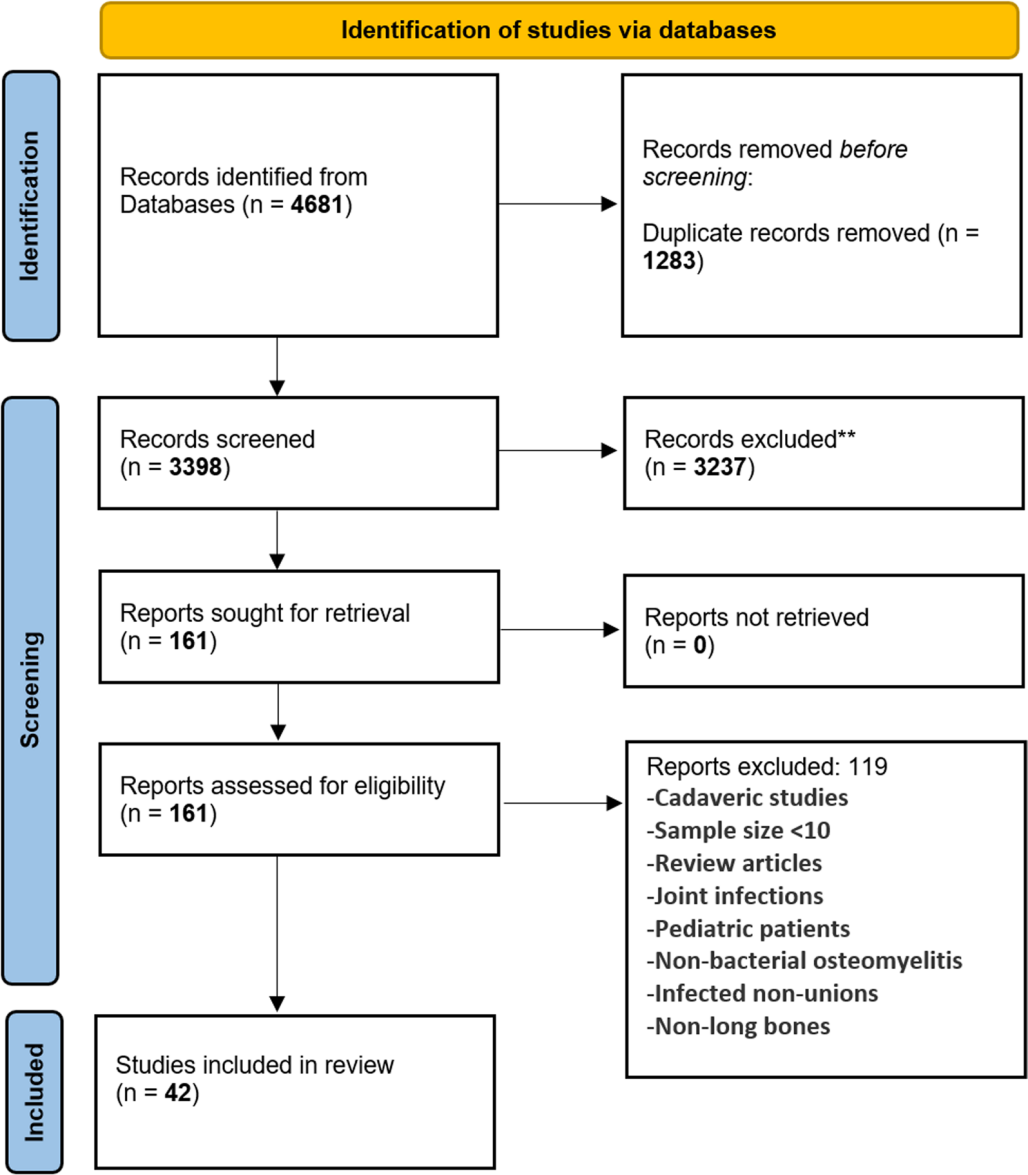
PRISMA flowchart illustrating inclusion of studies into the review

After the removal of duplicates from the initial search, a total of 3398 references were retrieved for title and abstract screening (Fig. 1). A total of 3237 articles were excluded after the initial title/abstract screening. Next, 161 studies underwent full-text review. A total of 42 studies were included in the final analysis.

### Sample data

The pooled patient demographics are outlined in Table 1. Among the included studies, all but one were retrospective, encompassing both cohort and retrospective comparative studies. A total of 1605 patients were analyzed, predominantly male (77%), with an average age of 42.7 ± 8.5 years. The mean follow-up duration was 36 ± 18 months. The studies predominantly focused on one-stage management (28 studies), while twelve opted for planned two-stage management, and two studies offered comparisons between single and two-stage management.

### Characteristics of osteomyelitis

The infection characteristics, host status, anatomical regions involved, and organisms are detailed in Table 2. The Cierny-Mader (CM) classification, reported in most studies, identified CM type III as the most common (60%) with host type B prevailing (51%). The etiology was primarily post-traumatic (64%, n = 812), followed by hematogenous origins (23%, n = 295). The tibia was the most affected site (57%, n = 819), with the femur (27%, n = 392) and humerus (6.8%, n = 98) following. Methicillin-susceptible Staphylococcus aureus (MSSA) was the predominant organism (28.5%, n = 357), with Methicillin-resistant Staphylococcus aureus (MRSA) found in 8.7% (109) of cases. Notably, 24.8% (n = 310) of cases showed no growth.

**Table 2:** Characteristics of the osteomyelitis according to classification, location and organisms

Study	Cierny Mader	Host type	Pathology	Bones	Organisms
Ochsner 1990 [30]	NR	NR	Post-traumatic 11, Hematogenous 1	Femur 7, Tibia 5	MSSA 7, Mixed 2, Pseudomonas 2, Coag-ve staph 1
Philandrianos 1992 [31]	NR	NR	Post traumatic 8, Contiguous 2	Tibia 5, femur 4, phalanx 1	MSSA 6, Coag-ve staph 1, proteus 1, E.coli 1, Salmonella 1
Guelinckx 1995 [32]	NR	NR	NR	Tibia 10	NR
Pfeiffenberger 1996 [33]	NR	NR	NR	Humerus 5	MSSA 4, No growth/unknown 1
Yamashita 1998 [34]	NR	NR	Hematogenous 12, Post-traumatic 4, iatrogenic 2	Tibia 9, humerus 2, femur 2	MSSA 4, MRSA 2, No growth/unknown 3, coag-'ve staph 1, Pseudomonas 2, streptococcus 1
Simpson 2001 [35]	CMI 2, CMII 3, CMIII 28, CMIV 8	A: 21, B: 16, C: 4	Hematogenous: 8, Post-traumatic: 29, Contiguous 4	Tibia 17, femur 16, humerus 5, radius 1, metatarsal 1, phalanx 1	MRSA 2, Coag-'ve staph 5, Mixed 15, MSSA 15, Diptheroids 1, Streptoccous 2, Proteus 1,
Kuokkanen 2002 [36]	NR	NR	Post-traumatic 16	Tibia 16	NR
Hashmi 2004 [37]	CMIII 6, CMIV: 11	A:16, B:1	Post-traumatic 8	Femur 4, Tibia 4	MSSA 4, Mixed 1, Coag- ‘ve staph 1, Pseudomonas 2
Chang 2007 [38]	CMI: 44, CMII: 2, CMIII: 16, CMIV: 3	A 55, B 9, C 1	Hematogenous 41, iatrogenic 13, post-traumatic 11	Femur 26, tibia 32, humerus 5, radius/ulna 2	MSSA 22, Pseudomonas 7, MRSA 6, Coag- ‘ve staph 4, Enterobacter cloacae 2, Micrococcus 1, No growth/unknown 23
Rao 2007 [39]	NR	NR	NR	tibia 3, tibia/fibula 2, femur 1, leg 1	VRE 3, coag-ve staph 2, Mixed 1, MSSA 1
Khan 2012 [40]	NR	NR	Post-traumatic 20	Tibia 20	NR
Romanò 2014 [41]	CMI 21, CMII 4, CMIII 46, CMIV 5	A: 29, B: 44, C: 3	Iatrogenic 31, post-traumatic 25, Hematogenous 20	Tibia 37, Femur 25, Humerus 3, Tibia/femur 1, Other 1	MRSA 28, MSSA 20, No growth/unknown 14, Coag-ve staph 13, Mixed 13, Enterococcus 8, Pseudomonas 8, Strep 2
Ferrando 2017 [26]	NR	NR	Iatrogenic 15, post-traumatic 8, Hematogenous 2	Tibia 13, femur 6, humerus 1	MSSA 11, MRSA 3, Pseudomonas 6, E.Coli 1, Finegoldia magna 1, streptococcus 1, Mixed 1
Badie 2019 [42]	CM I to III	C excluded	Hematogenous 17, post-traumatic 13	Ulna 1, radius 2, humerus 2, femur 11, tibia 14	MSSA 15, MRSA 3, Klebsiella 2, E.Coli 2, Proteus 2, Salmonella 1, Streptococcus 1, mixed 2, No growth/unknown 2
Oosthuysen 2019 [43]	CMIII 14	A: 7, B: 7	Post-traumatic 9, Hematogenous 4, Contiguous 1	Femur 3, Tibia 8, Radius 1, ulna 1, humerus 1	MSSA 3, No growth/unknown 3, Mixed 2, MRSA 2, Bifidobacterium 1, Enterobacter 1, Pseudomonas 1, Strep 1
Zhou 2020 [27]	CMIII 43	A: 36, BS:6, BL: 1	post-traumatic 31, hematogenous 10, contiguous 2	Tibia 43	No growth/unknown 22, MSSA 11, Enterococcus 2, Pseudomonas 2, Acinetobacter baumannii 1, Aeromonas hydrophilia 1, Coag-ve staph 1, E.coli 1, Klebsiella 1, Mixed 1
Al-Mousawi 2020 [44]	CMII 7, CMIII 5	NR	Post-traumatic 9, Contiguous 3	Femur 3, tibia 7, fibula 2	S.aureus 8, E.coli 3
Hotchen 2020 [45]	NR	NR	NR	Tibia 34, Femur 22, Humerus 8, Fibula 3, Radius 3, ulna 1	NR
Lorentzen 2020 [46]	NR	NR	Post-traumatic 8, iatrogenic 1	tibia 5, humerus 1, fibula 1, tibia/fibula 1, ulna 1	NR
Bor 2022 [47]	CMI 2, CMIII 12, CMIV 1	A: 9, B: 6	Post-traumatic 9, Hematogenous 3, iatrogenic 3	Tibia 7, Femur 4, humerus 2, fibula 1	Unspecified 4, MSSA 8, Pseudomonas 1, Serratia 1, Provedencia rettgeri 1
Elhessy 2022 [48]	CMI 14	A: 6, B: 8	NR	Tibia 11, femur 3	MRSA 8, MSSA 2, Pseudomonas 1, No growth/unknown 3
Jagadeesh 2022 [13]	CMI 21, CMIII 70, CMIV 7	A: 74, B: 26	NR	Tibia 74, femur 22, humerus 3, radius/ulna 1	No growth/unknown 30, mixed 9, MSSA 21, MRSA 9, Staph epidermidis 5, E.coli 4, Pseudomonas 7, others 15
Luo 2022 [49]	CMIII 11, CMIV 5	NR	Post-traumatic 16	Fibula 16	NR
McNally 2022 [24]	CMIII: 72, CMIV: 18	A: 19, B: 71	post-traumatic 71, hematogenous 19, iatrogenic 6, contiguous 4	Tibia 38, femur 24, humerus 16, radius/ulna 10, femur/tibia 1, fibula 1	MSSA 30, Mixed 21, Pseudomonas 7, Enterococcus 6, MRSA 6, Coag-ve staph 5, Enterobacter 5, E.Coli 5, Strep. 5, Cornyebacteria 4, Klebsiella 4, Proteus 4, Achromobacter 3, Morganella morganii 3, Bacillus 2, Citrobacter 2, Bacteroides 1, Clostridia 1, Propionobacter 1, Salmonella 1, Serratia 1
Jiamton 2023 [50]	CMI 11, CMII 3, CMIII 37, CMIV 11	A: 47, B: 15	Post-traumatic 48, hematogenous 14	Tibia 34, femur 19, humerus 4, calcaneus 2, clavicle 1, forearm 1, fibula 1	No growth/unknown 28, Mixed 7, Pseudomonas 9, MSSA 4, MRSA 1, Aerococcus viridans 1, Coag-'ve staph 2, Enterobacter 1, Enterococcus 1, Serratia 1, Staph cohnii 1, staph hemolyticus 1, staph hominis 1
Langit 2023 [51]	CMI 10, CMIII 39, CMIV 5	A: 23, B: 31	Post-traumatic 46, hematogenous 7, iatrogenic 1	Tibia 27, femur 10, humerus 9, fibula 5, ulna 2, radius 1	Mixed 12, No growth/unknown 13, MSSA 19, Enterobacter 2, salmonella 1, coag-ve staph 2, streptococcus 1, staph mitis 1, staph lugdunesis 1, anaerobes 1, pseudomonas 1
Sambri 2023 [52]	CMI: 31, CMII 13, CMIII 21, CMIV 28	A: 67, B: 26	Post-traumatic 25, Hematogenous 47, iatrogenic 21	Femur 24, Tibia 52. Humerus 6, radius 4, others 7	Negative 32, MRSA 18, MSSA 21, mixed 5, coag-'ve staph 10, enterobacter 7
Ferguson 2023 [14]	CMIII 284, CMIV 75	A: 99, B: 260	Post-traumatic 222, Hematogenous 83, iatrogenic 43, contiguous 11	Tibia 165, Femur 101, forearm 20, foot 11, Others 33	Mixed 73, No growth/unknown 106, MSSA 90, MRSA 12, coag-'ve staph 21, Pseudomonas 16, E.Coli 4, Enterobacter 8, Diphtheroids 2, Enterococcus 2, Proteus 3, Candida 2, Klebsiella 1, Bacteroids 1, Mycobacterium 3, Corynebacterium 3, serratia 3, strep 2, achromobacter 2, bacillus 2, salmonella 2, Cutibacterium acne 1, C.difficile 1
Perry 1986 [53]	NR	NR	Hematogenous 3, post-traumatic 5	Tibia 3, femur 4, radius 1	MSSA 5, Mixed 3
McNally 1993 [54]	NR	NR	post-traumatic 31, haematogenous 5, iatrogenic 1	Tibia 25,, femur 9, radius 2, humerus 1	NR
Ueng 1994 [25]	CMIII: 5	A: 5	Post-traumatic 5	Tibia 5	mixed 2, pseudomonas 2, serratia 1
Emara 2002 [29]	CMIII: 8, CMIV: 12	A:20	Post-traumatic 19, Hematogenous 1	Tibia 20	NR
Alonge 2003 [55]	NR	NR	Post-traumatic 4, Iatrogenic 5, Contiguous 2, NR 9	Tibia 7, femur 10, Humerus 1, ulna 2	MSSA 8, No growth/unknown 10, Proteus 2
Zweifel-Schlatter 2006 [56]	CMIII:10	B: 10	Post-traumatic 10	Tibia 10	MSSA 8, mixed 2, No growth/unknown 2
Wu 2007 [57]	CMIII 7	A: 6, B:1	Post-traumatic 7	Femur 7	No growth/unknown 1, Pseudomonas 1, Mixed 2, acinetobacter 1, enterobacter 1, MRSA 1,
Wu 2017 [11]	CMIV 36	B: 36	post-traumatic 35, hematogenous 1	femur 19, tibia 16, fibula 1	Mixed 13, MSSA 12, No growth/unknown 6, MRSA 5
Yu 2017 [9]	NR	NR	NR	Femur 13	Mixed 3, No growth/unknown 3, Citrobacter 1, MRSA 6
Qiu 2017 [10]	NR	NR	Post-traumatic 40	Tibia 40	MSSA 14, Coag-ve staph 3, Strept 2, Enterococcus 4, enterobacter 4, E.Coli 3, Klebsiella 3, Proteus 1, Pseudomonas 1, Citrobacter 1, acinetobacter 3,
Buono 2018 [58]	CMIII or IV: 24	NR	post-traumatic 23, iatrogenic 1	Tibia 24	NR
Wu 2019 [59]	CMI 8, CMIII 11, CMIV 9	A: 8, B: 20	post-traumatic 12, hematogenous 16	Humerus 28	MSSA 14, No growth/unknown 8, MRSA 6
Finelli 2019 [60]	CMI: 45	NR	Post-traumatic 45	tibia 29, femur 16	MSSA 23, Coag-ve staph 13, Enterococcus 5, Enterobacter 4, Strep 4, pseudomonas 3, Klebsiella 2, proteus 1, Providencia 1, serratia 1
Zhou 2021 [12]	NR	NR	NR	Tibia 76, femur 26	NR
Aggregates	CMI: CMII: 32 (2.9%) 207 (18.6%) CMIII: 667 (60%) CMIV: 205 (18.5%)	A: 547 (47.6%) B: 594 (51.7%) C: 8 (0.7%)	Post-traumatic: 812 (63.8%) Hematogenous: 295 (23.2%) Contiguous: 29 (2.8%) Iatrogenic: 137 (10.8%)	Tibia: 819 (57.2%) Femur: 392 (27.4%) Humerus: 98 (6.8%) Radius/ulna: 23 (1.6%) Fibula: 27 (1.9%) Others: 73 (5%)	MSSA: 357 (28.5%) MRSA: 109 (8.7%) Pseudomonas 66 (5.3%) Coag—‘ve staph: 85 (6.8%) Enterococcus: 22 (1.8%) Klebsiella: 9 (0.7%) Enterobacter: 30 (2.4%) E.coli: 18 (1.4%) Polymicrobial: 163 (13%) No growth/unknown: 310 (24.8%) Others: 83 (6.6%)

MSSA: Methicillin-Sensitive Staphylococcal Aureus, Methicillin-Resistant Staphylococcus Aureus

### Management strategies

The surgical treatment strategies are categorized in Table 3, including debridement, dead space management, soft tissue coverage, bone graft, and osseous stabilization. Dead space management techniques varied, with antibiotic-loaded calcium sulphate (CaSO4) beads (e.g., Stimulan, Osteoset T) used in 30.4% (n = 469) of cases. Polymethyl methacrylate (PMMA) cement was utilized in 15% (n = 236) of cases, employed as beads, spacers, and in Masquelet techniques. Other treatments included Cerament G (CaSO4 + hydroxyapatite), S53P4 bioactive glass, and others as described in Table 3. Flaps were required in 21.6% (n = 332) of cases, and bone grafts were used in 17% (n = 274), incorporating autologous, allograft, and reamer aspirate autograft.

**Table 3:** Management strategies and cure rates within the included studies

	Study	Management Protocol	Cure rate	Dead space Mx	Flaps	Bone graft
Onestage	Ochsner 1990 [30]	IM reaming + Abx loaded PMMA beads	7/8 (87.5%)	PMMA beads 8	None	None
		IM reaming + Suction irrigation drainage	4/4 (100%)			
	Philandrianos 1992 [31]	Debridement + Laser sterilisation + suction drainage + ABx loaded haemostatic device ± flap	10/10 (100%)	hemostatic device 9	local skin 4, Muscle 1	None
	Guelinckx 1995 [32]	Debridement + free muscle flap	10/10 (100%)	-	None	None
	Pfeiffenberger 1996 [33]	Debridment + Abx loaded PMMA beads	2/3 (66.6%)	PMMA beads 3	None	None
		Debridement only	1/2 (50%)			
	Yamashita 1998 [34]	Debridement + Abx loaded calcium-hydroxyapatite	13/13 (100%)	Ca-HA ceramic blocks 13	None	None
	Simpson 2001 [35]	Debridement + Abx loaded PMMA beads	24/30 (80%)	PMMA beads 30	Free 3, local 1, free fibula 1	None
		Debridement + free or local flap	3/3 (100%)			
		Minimal Debridement: drainage, tissue debulking, removal of sequestra and lavage	0/4 (0%)			
	Kuokkanen 2002 [36]	Debridement + muscle flap	15/16 (93.8%)	-	Muscle 16	3
		Debridement + bone graft + muscle flap	3/3 (100%)			
	Hashmi 2004 [37]	Debridment + IM suction irrigation drainage (Lautenbach technique)	8/8 (100%)	-	None	None
	Chang 2007 [38]	Debridement only	24/40 (60%)	CaSO4 beads 65	None	None
		Debridement + ABx loaded CaSO4	20/25 (80%)			
	Rao 2007 [39]	Debridement + Abx loaded PMMA beads	7/7 (100%)	PMMA beads 7	None	None
	Khan 2012 [40]	Debridement + free radial forearm fasciocutaneous flap	12/12 (100%)	-	Fasciocutaneous 20	None
		Debridement + autogenic bone graft + free radial forearm fasciocutaneous flap	8/8 (100%)			
	Romanò 2014 [41]	Debridement + hydroxyapatite & CaSO4	24/27 (99.9%)	CaSO4 beads 27, tricalcium PO4 beads 22, Bioglass 27	None	Bone matrix 22
		Debridement + s53p4 bioglass	25/27 (92.6%)			
		Debridement + tricalcium phosphate & ABx-loaded demineralised bone matrix	19/22 (86.4%)			
	Ferrando 2017 [26]	Debridement + Reamer-Irrigator-Aspirator + s53p4 bioglass	9/9 (100%)	CaSO4 beads 9, Bioglass s53p4 11	Muscle 3	None
		Debridement + Reamer-Irrigator-Aspirator + s53p4 bioglass + ALT flap	2/2 (100%)			
		Debridement ± Reamer-Irrigator-Aspirator + CaSO4 ± ALT flap	8/9 (89%)			
	Badie 2019 [42]	Debridement + Abx loaded CaSO4 mixed with bone marrow aspirate autograft ± flap	23/30 (77%)	CaSO4 beads 30	None	Bone marrow aspirate 30
	Oosthuysen 2019 [43]	Debridment + s53p4 bioglass	13/14 (92.9%)	Bioglass 14	None	None
	Zhou 2020 [27]	Debridement + Abx loaded CaSO4	37/41 (90.2%)	CaSO4 beads 43	Unspecified 2	None
		Debridement + Abx loaded CaSO4 + flap	1/2 (50%)			
	Al-Mousawi 2020 [44]	Debridment + keystone perforator island flap	11/12 (91.7%)	-	Fasciocutaneous 12	None
	Hotchen 2020 [45]	Debridement ± (Abx loaded CaSO4 with CaCO3) OR (abx loaded CaSO4 with hydroxyapatite) ± flap	61/63 (96.8%)	CaSO4 + CaCO3 beads, CaSO4 beads + HA. Numbers NR	Unspecified 54	None
	Lorentzen 2020 [46]	Debridement + Abx loaded CaSO4 with hydroxyapatite ± flap	8/9 (88.9%)	CaSO4 + HA 9	Muscle 9	None
	Bor 2022 [47]	Debridement + removal of metal + Abx loaded PMMA cement	15/15 (100%)	PMMA beads 1, IM nail/rod 3, cemented rod 2, Cement blocks 8	None	None
	Elhessy 2022 [48]	Debridement + IM reaming and irrigation + Abx loaded CaSO4	14/14 (100%)	CaSO4 beads 14	None	None
	Jagadeesh 2022 [13]	Debridement + Abx loaded CaSO4	44/50 (88%)	CaSO4 pellet 50	Unspecified 10	50
		Debridement + bone graft	32/50 (64%)			
	Luo 2022 [49]	Debridement + distally based peroneal artery perforator + fasciocutaneous flap	16/16 (100%)	-	Fasciocutaneous 16	None
	McNally 2022 [24]	Abx loaded CaSO4 hydroxyapatite ± flap	66/70 (94.3%)	CaSO4 + HA 70	NR*	None
	Jiamton 2023 [50]	Debridement + Abx loaded microporous nanohydroxyapatite (nHA-ATB) beads ± flap	52/53 (98.11%)	Nanohydroxyapatite beads 62	None	None
	Langit 2023 [51]	Debridement ± IM reaming & irrigation + stimulan or cerament G ± flap	45/53 (85%)	-	Unspecified 11	None
	Sambri 2023 [52]	Debridement + PerOssal beads ± flap	70/93 (74.5%)	PerOssal beads 93	local 2, free 5	None
	Ferguson 2023 [14]	Debridement + osteoset T ± flap	159/179 (88.8%)	CaSO4 beads 179, CaSO4 + HA 180	Muscle 98	None
		Debridement + cerament G ± flap	172/180 (96.6%)			
Two stage	Perry 1986 [53]	**Stage 1:** Debridement + Abx loaded implantable pump.** Stage 2:** pump removal	5/8 (62.5%)	Implantable pump 8	None	None
	McNally 1993 [54]	**Stage 1:** Debridement + Abx loaded PMMA beads or muscle flap;** Stage 2:** redebridment ± removal of beads + autogenous bone transplant	28/32 (92%)	PMMA beads 23	Muscle 14	37
	Ueng 1994 [25]	**Stage 1:** Debridment + Abx PMMA beads;** Stage 2:** Removal of beads, autogenous bone graft	5/5 (100%)	PMMA beads: 5	None	5
	Emara 2002 [29]	**Stage 1:** Debridement + corticotomy;** Stage 2:** corticotomy + Segment transfer	19/20 (95%)	-	None	None
	Alonge 2003 [35]	**Stage 1:** Debridement + Abx loaded PMMA beads + flap;** Stage 2:** redebridment + removal of beads + autogenous bone graft	17/20 (85%)	PMMA beads 19 (3 patients had 1 stage with retained PMMA)	Fasciocutaneous 2, cross-leg 1	3
	Zweifel-Schlatter 2006 [56]	**Stage 1:** Debridement ± continous irrigation/drainage;** Stage 2:** Debridement + free fasciocutaneous flap	6/6 (100%)	-	Fasciocutaneous 10	4
		**Stage 1:** Debridement ± continous irigation/drainage; **Stage 2:** Debridement + free fasciocutaneous flap + bone graft	4/4 (100%)			
	Wu 2007 [57]	**Stage 1:** Debridement + Abx loaded PMMA beads;** Stage 2**: plate + bone graft	6/7 (85.7%)	PMMA beads 7	None	7
	Wu 2017 [11]	**Stage 1:** Debridement + Abx PMMA spacer ± flap;** Stage 2:** Removal of spacer, bone graft	30/36 (83.3%)	PMMA powder/spacer 36	Unspecified 8	36
	Yu 2017 [9]	**Stage 1:** Debridement, plate, PMMA spacer;** Stage 2:** removal of spacer, bone graft (Masquelet)	12/13 (92.3%)	PMMA spacer 13	None	13
	Qiu 2017 [10]	**Stage 1:** Debridement + Abx loaded PMMA beads;** Stage 2:** beads removal + bone graft	16/18 (88.9%)	PMMA beads 18, Cement Spacer 22	Fasciocutaneous 18	40
		**Stage 1:** Debridement + Abx loaded spacer (Masquelet);** Stage 2:** spacer removal + bone graft	20/22 (90.9%)			
	Buono 2018 [58]	**Stage 1:** Debridement + Abx loaded PMMA beads;** Stage 2:** Bead removal + bone graft + free muscle flap	11/13 (84.6%)	PMMA beads 24	Muscle 13, Fasciocutaneous 11	11
		**Stage 1:** Debridement + Abx loaded PMMA beads;** Stage 2:** Bead removal + bone graft + free fasciocutaneous flap	10/11 (90.9%)			
	Wu 2019 [59]	**Stage 1:** Debridement + Abx rod;** Stage 2:** removal of cement rod ± masquelet bone grafting	5/8 (62.5%)	Cemented rod 8, PMMA spacer 20	None	13
		**Stage 1:** Debridement + Abx PMMA spacer;** Stage 2:** removal of spacer ± masquelet bone grafting	20/20 (100%)			
Compared	Finelli 2019 [60]	Debridment + IM reaming with Reamer Irrigator Aspirator	20/23 (87%)	One-stage: None Two-stage: PMMA spacer 22	None	None
		**Stage 1:** Debridement + Conventional IM reaming + Abx PMMA spacer;** Stage 2:** removal of spacer	21/22 (95.5%)			
	Zhou 2021 [12]	Debridement + Abx loaded CaSO4 + osteotomy + bone transport	61/70 (87.1%)	Total: CaSO4 implantation 102	None	None
		**Stage 1:** Debridement + Abx loaded CaSO4;** Stage 2:** osteotomy + bone transport	30/32 (93.8%)			
	Aggregates			**PMMA implant:** 236 (15.7%) **CaSO4 beads (osteoset T, Stimulan):** 469 (31.2%) **S53P4 Bioglass:** 52 (3.5%) **CaSO4 + HA (Cerament G):** 259 (17.2%) **PerOssal beads:** 93 (6.2%) **Nanohydroxyapatite beads:** 62 (4.1%) **Others:** 145 (9.6%) **None:** 188 (12.5%)	**Total flap use:** 332 (21.6%) **muscle flaps:** 154 (10%) **Fasciocutaneous flaps:** 89 (5.8%) **Unspecified/others:** 89 (5.8%)	**Total Bone graft use:** 274 (17%)

*Unable to exclude cases not meeting criteria. NR, Not reported

### Complications

Complications reported across studies exhibited considerable heterogeneity, detailed in Table 4. Recurrence of infection was treated as a failure, not a complication, and is thus analyzed separately under cure rates. The overall complication rates were similar for both single-stage and two-stage treatments (26.6% and 27.6%, respectively). The most frequent complication in single-stage procedures was prolonged wound drainage (13%), with stiffness and reduced range of motion also commonly reported.

**Table 4:** Aggregated complications in both groups

Complication	One stage	Two stages
Chronic pain	8	0
Complete flap failure/anastomosis thrombosis managed surgically	2	3
Thromboembolism (DVT, PE)	3	0
Fracture managed surgically	17	2
Hematoma managed surgically	1	2
Hematoma managed conservatively	1	0
Non-union/mail-union	11	7
Partial flap failure managed conservatively	2	0
Partial flap failure managed surgically	2	0
Flap edema	1	0
Pin site infection	17	19
Prolonged wound leakage	150	10
Reduced range of motion	35	24
Reduced sensation/nerve injury	19	1
Seroma	1	0
Abscess	1	0
Wound healing problems/superficial infections managed surgically	13	4
Wound healing problems/superficial infections managed conservatively	7	0
Deep infection managed conservatively	4	0
Acute on top of chronic osteomyelitis	1	0
Amputation	3	1
Kidney failure	2	0
Skin rash	0	1
Unrelated/insignificant Complications	20	0
Overall complication rate (Excluding death due to disease, recurrence of COM and unrelated/insignificant Cx)	301/1131 (26.6%)	74/268 (27.6%)

### Meta-analysis of cure rates

**Fig. 2:**
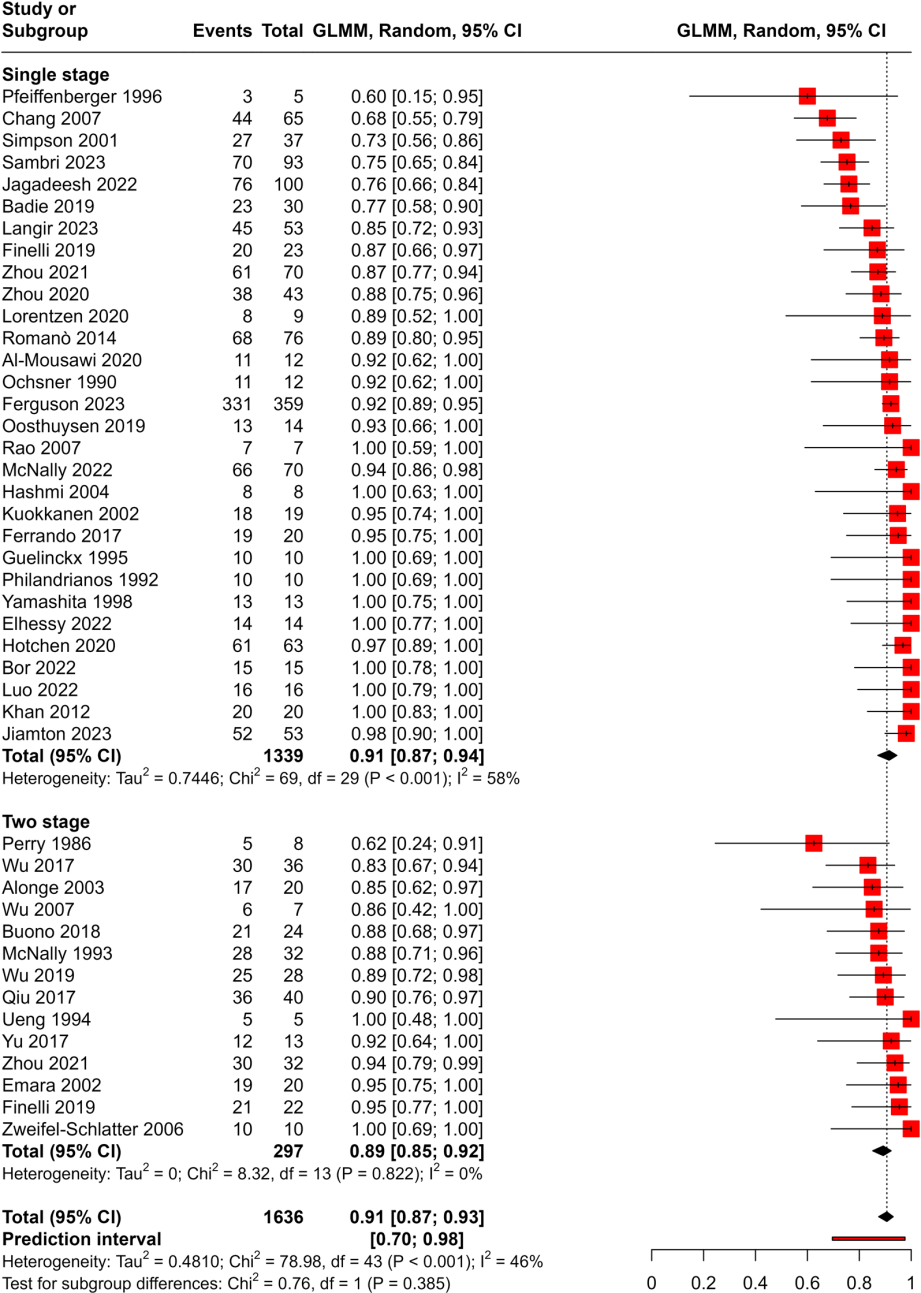
Meta-analysis of cure rates in single and two-stage groups

The analysis included 1636 patients. Single stage method was used in 1339 patients and the two-stage method was used in 297 patients. The pooled cure rate was 91% (CI 87%; 93%). Stratifying the analysis by stage did not reveal a statistically significant difference (X^2^ = 0.76, P > 0.05) with similar cure rate across stages (Fig. 2). The funnel plot was symmetric indicating the absence of publication bias. Egger’s test was not statistically significant (P = 0.64).

## Discussion

In our systematic review and meta-analysis on the treatment of osteomyelitis, we examined the evolving therapeutic strategies for this complex condition. Our findings reveal that, in terms of cure rates, or more appropriately termed, non-recurrence rates, there appears to be no significant difference when comparing single versus two-stage management of chronic osteomyelitis. This analysis is the first to collectively assess the success rates of single versus two stage management.

The decision between single-stage and multi-stage procedures is important, particularly considering the implications of lengthier hospital stays, increased costs, and operational complexities associated with two-stage management. Zhou et al. highlighted the notably higher costs and extended hospital stays associated with two-stage procedures compared to single-stage management [12]. Their findings indicate an average hospital stay of 28 days for the two-stage group, versus 18 days for those undergoing single-stage procedures. Similar trends are noted in studies by McNally, Ueng, and Qiu, reporting hospital stays of 27, 22, and 24 days respectively in two-stage treatment [10, 24, 25]. However, variability in hospital stay lengths is influenced by different institutional protocols and the possibility of outpatient management. Across studies, a comprehensive report on the costs and durations of hospital stays is generally deficient.

The surgical aspect of treatment is intricate, and our data indicates that debridement alone is associated with lower cure rates. Quantifying the extent of debridement in various studies presents another challenge, as the terminology used to describe it, such as "radical" or "adequate," is open to diverse interpretations. Consequently, the current data does not allow for distinct categorization of debridement methods.

Dead space management has become increasingly significant in recent years. Techniques such as antibiotic-coated beads and cement, muscle flaps, and bone grafts for addressing compromised soft tissue and bone loss have shown favorable outcomes based studies included in this review.. The induced membrane or Masquelet technique, though requiring a two-stage approach, has shown reliable results in our review [10, 11]. Additionally, bone defect management techniques, such as circular frames and bone segment transfers, offer stability, enabling early range of motion and weight-bearing. Jagadeesh et al.'s study reported a higher success rate with the use of calcium sulfate compared to debridement alone [13]. The current evidence suggests that the effectiveness of various local antibiotic delivery systems is comparable [14, 26].

Complication reporting varied across studies, with a notable incidence of prolonged wound drainage in single-stage procedures, often associated with calcium sulfate beads. While concerning, this drainage is not necessarily a harbinger of infection. Ferguson et al. reported high rates of wound leakage using calcium sulphate beads, but highlighted the low risk of infection associated with it [14]. Jagadeesh et al. also reported 18 out of 50 patients had ongoing serous discharge with the use of calcium sulphate that lasted up to 4 weeks resolving without treatment other than dressing changes [13]. This is in line with reports of 4–30% serous discharge while calcium sulphate is undergoing resorption. It is perhaps mitigated by adequate soft tissue coverage and judicious use of calcium sulphate [12, 14, 27, 28]

Commonly reported complications included wound issues, stiffness, and neuropathic symptoms, which could potentially be alleviated by early rehabilitation following extensive surgeries. Moreover, data on postoperative range of motion is scarce; improved reporting could reveal differences in single-stage groups, potentially allowing for earlier postoperative rehabilitation. More pin site issues were observed in the two-stage group, potentially due to longer durations of fixator use [10, 12, 29]. However, drawing definitive conclusions in this regard is difficult due to the more frequent use of fixators in two-stage management, as well as variations in the definition of pin-site infections. The occurrence of fractures in both treatment approaches necessitates cautious management, particularly regarding the introduction of implants. Systemic complications such as deep vein thrombosis, pulmonary embolism, and acute kidney injuries were also noted, albeit less frequently.

Perhaps it is key to highlight the literature's deficiencies, including the heterogeneity in antibiotic administration, inclusion criteria covering various bones and etiologies, and diverse causative organisms. These variations make it challenging to conclusively determine the superiority of specific treatments. In addition, the analysis represents single-arm comparisons, which are potential sources of bias. Although studies utilized similar techniques for both single and two-stage procedures, certain factors, such as the degree of osteomyelitis and the patient's physiological status, may indicate the use of one technique over the other. Another point of interest would be an analysis of the potential complications in both treatment groups. However, the variation in definitions of complications and the lack of clear reporting of complications arising due to disease and treatment did not allow for an accurate analysis in this regard.

Future research should focus on prospective studies, examining variables like causative organisms, patient demographics, Cierny-Mader classification, and specific treatment modalities. In addition, it will be helpful to know whether major differences exist between different preparations of antibiotic coated beads.

## Conclusion

Chronic osteomyelitis is a complex condition with various treatments and interventions described. The data from our analysis suggests that single and two-stage treatment of chronic osteomyelitis yields comparably effective results. The current treatment strategies included a combination of debridement, dead space management, local and systematic antibiotics along with bone stabilization and soft tissue reconstruction if necessary. However, the indications for using either technique may play a role in predicting success rates. Higher-level studies should be conducted to provide more generalizable conclusions.
