# Correction: Single versus two-stage management of long-bone chronic osteomyelitis in adults: a systematic review and meta-analysis

**DOI:** 10.1186/s13018-024-04976-6

**Published:** 2024-08-31

**Authors:** Ali Lari, Ali Esmaeil, Matthew Marples, Arun Watts, Bethan Pincher, Hemant Sharma

**Affiliations:** 1 Al-Razi Orthopedic Hospital, Kuwait, Kuwait; 2 Hull University Teaching Hospitals, Hull, UK; 3 Hull York Medical School, University of Hull, Hull Limb Reconstruction & Bone Infection Unit, Hull University Teaching Hospitals, Hull, UK

**Correction: Journal of Orthopaedic Surgery and Research (2024) 19:351** **https://doi.org/10.1186/s13018-024-04832-7**

There were several errors in formatting (including multiple articles in the same cell in the first column). NO changes were made to any content and only formatting and reordering was done to comply with original work. Citations are not in line with the content. Wu 2019 [59] has never published about PerOssal, the citation must be Sambri 2023 [52]. The content given for Sambri 2023 [52] is revised.

The original article has been corrected.

**Table 3:** Management strategies and cure rates within the included studies

	Paper	Management protocol	Cure rate	Dead space Mx	Flaps	Bone graft
One stage	Ochsner 1990 [30] Philandrianos 1992 [31]	IM reaming + Abx loaded PMMA beads	7/8 (87.5%)	PMMA beads 8	None	None
		IM reaming + Suction irrigation drainage	4/4 (100%)			
	Guelinckx 1995 [32]	Debridement + Laser sterilisation + suction drainage + ABx loaded haemostatic device ± flap	10/10 (100%)	hemostatic device 9	local skin 4, Muscle 1	None
	Pfeiffenberger 1996 [33]	Debridement + free muscle flap	10/10 (100%)	-	None	None
	Yamashita 1998 [34] Simpson 2001 [35]	Debridment + Abx loaded PMMA beads	2/3 (66.6%)	PMMA beads 3	None	None
		Debridement only	1/2 (50%)			
	Kuokkanen 2002 [36]	Debridement + Abx loaded calcium-hydroxyapatite	13/13 (100%)	Ca-HA ceramic blocks 13	None	None
	Hashmi 2004 [37] Chang 2007 [38] Rao 2007 [39]	Debridement + Abx loaded PMMA beads	24/30 (80%)	PMMA beads 30	Free 3, local 1, free fibula 1	None
		Debridement + free or local flap	3/3 (100%)			
		Minimal Debridement: drainage, tissue debulking, removal of sequestra and lavage	0/4 (0%)			
	Khan 2012[40] Romanò 2014 [41]	Debridement + muscle flap	15/16 (93.8%)	-	Muscle 16	3
		Debridement + bone graft + muscle flap	3/3 (100%)			
	Ferrando 2017 [26]	Debridment + IM suction irrigation drainage (Lautenbach technique)	8/8 (100%)	-	None	None
	Badie 2019 [42] Oosthuysen 2019 [43]	Debridement only	24/40 (60%)	CaSO4 beads 65	None	None
		Debridement + ABx loaded CaSO4	20/25 (80%)			
	Zhou 2020 [27]	Debridement + Abx loaded PMMA beads	7/7 (100%)	PMMA beads 7	None	None
	Al-Mousawi 2020 [44] Hotchen 2020 [45]	Debridement + free radial forearm fasciocutaneous flap	12/12 (100%)	-	Fasciocutaneous 20	None
		Debridement + autogenic bone graft + free radial forearm fasciocutaneous flap	8/8 (100%)			
	Lorentzen 2020 [46] Bor 2022 [47] Elhessy 2022 [48]	Debridement + hydroxyapatite & CaSO4	24/27 (99.9%)	CaSO4 beads 27, tricalcium PO4 beads 22, Bioglass 27	None	Bone matrix 22
		Debridement + s53p4 bioglass	25/27 (92.6%)			
		Debridement + tricalcium phosphate & ABx-loaded demineralised bone matrix	19/22 (86.4%)			
	Jagadeesh 2022 [13] Luo 2022 [49] McNally 2022 [24]	Debridement + Reamer-Irrigator-Aspirator + s53p4 bioglass	9/9 (100%)	CaSO4 beads 9, Bioglass s53p4 11	Muscle 3	None
		Debridement + Reamer-Irrigator-Aspirator + s53p4 bioglass + ALT flap	2/2 (100%)			
		Debridement ± Reamer-Irrigator-Aspirator + CaSO4 ± ALT flap	8/9 (89%)			
	Jiamton 2023 [50]	Debridement + Abx loaded CaSO4 mixed with bone marrow aspirate autograft ± flap	23/30 (77%)	CaSO4 beads 30	None	Bone marrow aspirate 30
	Langit 2023 [51]	Debridment + s53p4 bioglass	13/14 (92.9%)	Bioglass 14	None	None
	Sambri 2023 [52] Ferguson 2023 [14]	Debridement + Abx loaded CaSO4	37/41 (90.2%)	CaSO4 beads 43	Unspecified 2	None
		Debridement + Abx loaded CaSO4 + flap	1/2 (50%)			
	Perry 1986 [53]	Debridment + keystone perforator island flap	11/12 (91.7%)	-	Fasciocutaneous 12	None
	McNally 1993 [54]	Debridement ± (Abx loaded CaSO4 with CaCO3) OR (abx loaded CaSO4 with hydroxyapatite) ± flap	61/63 (96.8%)	CaSO4+CaCO3 beads, CaSO4 beads + HA. Numbers NR	Unspecified 54	None
	Ueng 1994 [25]	Debridement + Abx loaded CaSO4 with hydroxyapatite ± flap	8/9 (88.9%)	CaSO4 +HA 9	Muscle 9	None
	Emara 2002 [29]	Debridement + removal of metal + Abx loaded PMMA cement	15/15 (100%)	PMMA beads 1, IM nail/rod 3, cemented rod 2, Cement blocks 8	None	None
	Alonge 2003 [55]	Debridement + IM reaming and irrigation + Abx loaded CaSO4	14/14 (100%)	CaSO4 beads 14	None	None
	Zweifel-Schlatter 2006 [56] Wu 2007 [57]	Debridement + Abx loaded CaSO4	44/50 (88%)	CaSO4 pellet 50	Unspecified 10	50
		Debridement + bone graft	32/50 (64%)			
	Wu 2017 [11]	Debridement + distally based peroneal artery perforator + fasciocutaneous flap	16/16 (100%)	-	Fasciocutaneous 16	None
	Yu 2017 [9]	Abx loaded CaSO4 hydroxyapatite ± flap	66/70 (94.3%)	CaSO4 +HA 70	NR*	None
	Qiu 2017 [10]	Debridement + Abx loaded microporous nanohydroxyapatite (nHA-ATB) beads ± flap	52/53 (98.11%)	Nanohydroxyapatite beads 62	None	None
	Buono 2018 [58]	Debridement ± IM reaming & irrigation + stimulan or cerament G ± flap	45/53 (85%)	-	Unspecified 11	None
	Wu 2019 [59]	Debridement + PerOssal beads ± flap	70/93 (74.5%)	PerOssal beads 93	local 2, free 5	None
	Finelli 2019 [60] Zhou 2021 [12]	Debridement + osteoset T ± flap	159/179 (88.8%)	CaSO4 beads 179, CaSO4 + HA 180	Muscle 98	None
		Debridement + cerament G ± flap	172/180 (96.6%)			
Two stage	Perry 1986	**Stage 1**: Debridement + Abx loaded implantable pump. **Stage 2**: pump removal	5/8 (62.5%)	Implantable pump 8	None	None
	McNally 1993	**Stage 1**: Debridement + Abx loaded PMMA beads or muscle flap; **Stage 2:** redebridment ± removal of beads + autogenous bone transplant	28/32 (92%)	PMMA beads 23	Muscle 14	37
	Ueng 1994	**Stage 1**: Debridment + Abx PMMA beads; **Stage 2**: Removal of beads, autogenous bone graft	5/5 (100%)	PMMA beads: 5	None	5
	Emara 2002	**Stage 1**: Debridement + corticotomy; **Stage 2**: corticotomy + Segment transfer	19/20 (95%)	-	None	None
	Alonge 2003	**Stage 1**: Debridement + Abx loaded PMMA beads + flap; **Stage 2**: redebridment + removal of beads + autogenous bone graft	17/20 (85%)	PMMA beads 19 (3 patients had 1 stage with retained PMMA)	Fasciocutaneous 2, cross-leg 1	3
	Zweifel-Schlatter 2006	**Stage 1**: Debridement ± continous irrigation/drainage; **Stage 2**: Debridement + free fasciocutaneous flap	6/6 (100%)	-	Fasciocutaneous 10	4
		**Stage 1**: Debridement +/- continous irigation/drainage; **Stage 2**: Debridement + free fasciocutaneous flap + bone graft	4/4 (100%)			
	Wu 2007	**Stage1**: Debridement + Abx loaded PMMA beads; **Stage2**: plate + bone graft	6/7 (85.7%)	PMMA beads 7	None	7
	Wu 2017	**Stage 1**: Debridement + Abx PMMA spacer ± flap; **Stage 2**: Removal of spacer, bone graft	30/36 (83.3%)	PMMA powder/spacer 36	Unspecified 8	36
	Yu 2017	**Stage 1**: Debridement, plate, PMMA spacer; **Stage 2:** removal of spacer, bone graft (Masquelet)	12/13 (92.3%)	PMMA spacer 13	None	13
	Qiu 2017	**Stage1**: Debridement + Abx loaded PMMA beads; **Stage2**: beads removal + bone graft	16/18 (88.9%)	PMMA beads 18, Cement Spacer 22	Fasciocutaneous 18	40
		**Stage1**: Debridement + Abx loaded spacer (Masquelet); Stage 2: spacer removal + bone graft	20/22 (90.9%)			
	Buono 2018	**Stage 1**: Debridement + Abx loaded PMMA beads; **Stage 2**: Bead removal + bone graft + free muscle flap	11/13 (84.6%)	PMMA beads 24	Muscle 13, Fasciocutaneous 11	11
		**Stage 1**: Debridement + Abx loaded PMMA beads; **Stage 2**: Bead removal + bone graft + free fasciocutaneous flap	10/11 (90.9%)			
Compared	Wu 2019	**Stage 1**: Debridement + Abx rod; **Stage 2**: removal of cement rod ± masquelet bone grafting	5/8 (62.5%)	Cemented rod 8, PMMA spacer 20	None	13
		**Stage 1**: Debridement + Abx PMMA spacer; **Stage 2**: removal of spacer ± masquelet bone grafting	20/20 (100%)			
	Finelli 2019	Debridment + IM reaming with Reamer Irrigator Aspirator	20/23 (87%)	One-stage: None Two-stage: PMMA spacer 22	None	None
		**Stage 1**: Debridement + Conventional IM reaming + Abx PMMA spacer; **Stage 2**: removal of spacer	21/22 (95.5%)			
	Zhou 2021	Debridement + Abx loaded CaSO4 + osteotomy + bone transport	61/70 (87.1%)	Total: CaSO4 implantation 102	None	None
		**Stage 1**: Debridement + Abx loaded CaSO4; **Stage 2**: osteotomy + bone transport	30/32 (93.8%)			
	Aggregates			**PMMA implant**: 236 (15.7%) **CaSO4 beads (osteoset T, Stimulan):** 469 (31.2%) **S53P4 Bioglass:** 52 (3.5%) **CaSO4+HA (Cerament G)**: 259 (17.2%) **PerOssal beads**: 93 (6.2%) **Nanohydroxyapatite beads:** 62 (4.1%) **Others**: 145 (9.6%) **None**: 188 (12.5%)	**Total flap use:** 332 (21.6%) **muscle flaps**: 154 (10%) **Fasciocutaneous flaps**: 89 (5.8%) **Unspecified/others**: 89 (5.8%)	**Total Bone graft use**: 274 (17%)

